# Classification of Copper Minerals by Handheld Laser-Induced Breakdown Spectroscopy and Nonnegative Tensor Factorisation

**DOI:** 10.3390/s20185152

**Published:** 2020-09-09

**Authors:** Michał Wójcik, Pia Brinkmann, Rafał Zdunek, Daniel Riebe, Toralf Beitz, Sven Merk, Katarzyna Cieślik, David Mory, Arkadiusz Antończak

**Affiliations:** 1 Department of Field Theory, Electronic Circuits and Optoelectronics, Faculty of Electronics, Wroclaw University of Science and Technology, Wybrzeze Wyspianskiego 27, 50370 Wroclaw, Poland; michal.wojcik@pwr.edu.pl (M.W.); arkadiusz.antonczak@pwr.edu.pl (A.A.); 2 Physical Chemistry, University of Potsdam, Karl-Liebknecht-Str. 24-25, 14476 Potsdam, Germany; pbrinkma@uni-potsdam.de (P.B.); riebe@uni-potsdam.de (D.R.); beitz@uni-potsdam.de (T.B.); 3 LTB Lasertechnik Berlin GmbH, Am Studio 2c, 12489 Berlin, Germany; sven.merk@ltb-berlin.de (S.M.); katarzyna.cieslik@ltb-berlin.de (K.C.); david.mory@ltb-berlin.de (D.M.)

**Keywords:** LIBS, NTF, HALS, classification, copper minerals

## Abstract

Laser-induced breakdown spectroscopy (LIBS) analysers are becoming increasingly common for material classification purposes. However, to achieve good classification accuracy, mostly noncompact units are used based on their stability and reproducibility. In addition, computational algorithms that require significant hardware resources are commonly applied. For performing measurement campaigns in hard-to-access environments, such as mining sites, there is a need for compact, portable, or even handheld devices capable of reaching high measurement accuracy. The optics and hardware of small (i.e., handheld) devices are limited by space and power consumption and require a compromise of the achievable spectral quality. As long as the size of such a device is a major constraint, the software is the primary field for improvement. In this study, we propose a novel combination of handheld LIBS with non-negative tensor factorisation to investigate its classification capabilities of copper minerals. The proposed approach is based on the extraction of source spectra for each mineral (with the use of tensor methods) and their labelling based on the percentage contribution within the dataset. These latent spectra are then used in a regression model for validation purposes. The application of such an approach leads to an increase in the classification score by approximately 5% compared to that obtained using commonly used classifiers such as support vector machines, linear discriminant analysis, and the *k*-nearest neighbours algorithm.

## 1. Introduction

Laser-induced breakdown spectroscopy (LIBS) [1] is a remote sensing technique used for both qualitative and quantitative analysis of various materials. The operational principle is to use a high pulse energy laser to instantaneously heat the matter to evaporate a small amount of the substrate and eject it as a plasma plume. Then, the light emitted by the plasma is dispersed and registered by a camera. After the specified time of continuous wavelength radiation, a time window exists with the quickly cooling plasma, whereas the individual spectral lines representing the elemental material composition can be recorded.

The LIBS itself (apart from intensity of elements’ spectral lines) does not deliver analytically relevant information, such as classification or quantification. For these purposes, software methods must be engaged based on the input spectra and some mathematical operations to deliver the expected measurement outcome. The algorithms used for such spectral data analysis are numerous and cover a large area of statistics and machine learning fields of study [2,3]. Among them, the most frequently used with LIBS are: classifiers (linear discriminant analysis (LDA) [4], support vector machines (SVM) [5], and *k*-nearest neighbours (KNN) [6])); regression models (partial least squares [7], lasso [8], and Bayesian regression [9])); clustering algorithms (*k*-means [10]) and artificial neural networks (ANN)) [11].

There are numerous fields where LIBS analysers are used [12], such as basic scrap metal analysis [13], classification of alloys [14], or mapping of geological cores [15]. Additional uses include sophisticated applications such as adulteration detection in milk [16], discrimination of heavy-metal-contamination in seafood [17], analysis of pathological tissues [18], or precision agriculture [19]. They are even used with the most demanding space missions [20] and biohazard detection [21]. The size of the LIBS equipment can vary from small handheld devices to large workstations. The size is usually commensurate with the increase in resolution, limit of detection, repeatability, and general unit functionality. However, the device size limits its usage in many in-situ applications where a handy analyser is perfect. Such situations occur when the amount of equipment is limited by personal lifting capacity or where the measurement location is extremely tight (i.e., mines and other geological sites).

In this study, we focus on the possibility of using a compact handheld LIBS to analyse the geological samples online—on-site. The materials analysed will be copper minerals existing in a natural state on their base substrate, so no sample preparations will be made. The outcome for the measurements should be a mineral identification of the rocks exposed to laser radiation. However, this is a nontrivial task because the mineral samples are very heterogeneous with irregular geometrical shapes. This heterogeneity makes it difficult for classification learners as the labels may be incorrectly assigned or impure, leading to misclassifications between minerals. Conversely, the irregular mineral shape becomes challenging for the optics and lasing capabilities of the handheld device, and the registered spectra will differ significantly in intensity and spectral channel coverage. Our proposed approach is to use a linear regression model for the classification purpose, while the regressors originate from a blind source separation algorithm. This algorithm will be capable of distinguishing the mineral spectra of interest from the base rock or other impurities of the analysed sample. In this case, a novel combination of LIBS with a non-negative tensor factorisation (NTF) [22] method was applied.

The NTF is an unsupervised learning method for extracting mode-related non-negative latent components from a multiway array (tensor). Assuming that the measured LIBS spectra from multiple measurement points can be collected to a 3-way tensor, the aim of NTF is to extract a few artificial (latent) spectra from each mineral in a given dataset, and then use them as predictors in a regression model for classification purposes. The latent spectra can be regarded as common patterns in the observed LIBS spectra and have a multilinear relationship with the spectra across each mode of the observed tensor. Such multimodal relationships cannot be revealed with matrix factorisation models, such as principal component analysis (PCA). Moreover, due to nonnegativity constraints, NTF yields spectra that have a physical sense and easy interpretation, whereas PCA provides only some orthogonal components (with negative entries) that could not be used as predictors in a regression model.

The final intent of this work is to create an analytical method that could be embedded within handheld or other mobile LIBS devices or systems, which can be used as a support and verification tool for geologists while prospecting for copper deposits.

## 2. Materials

### 2.1. Overview

This work is based on the analysis of 62 different copper minerals whose copper content varies significantly. Those minerals are: **azu**rite, **mal**achite, **bro**chantite, **cop**iapite, **dev**illine, **for**nacite, **lan**gite, **nak**auriite, **nat**rochalcite, **osa**rizawaite, **pos**njakite, **vau**quelinite, **art**hurite, **che**nevixite, **cli**noclase, **con**ichalcite, cornetite (**co0**), **cor**nubite, **des**cloizite, **duf**tite, **lib**ethenite, **mot**tramite, **oli**venite, **par**nauite, **pse**udomalachite, **ric**helsdorfite, **tsu**mebite, **tur**quoise, **tyr**olite, **ajo**ite, **all**ophane, **chr**ysocolla, **cre**aseyite, **dio**ptase, **hal**loysite, **pla**ncheite, **ves**uvianite, **alg**odonite, **ant**imonpearceite, **bor**nite, **bou**rnonite, **cha**lcopyrite, **col**usite, **cov**ellite, **dig**enite, **ena**rgite, **fre**ibergite, **ger**manite, **gla**dite, **ida**ite, **jas**kolskiite, **kru**pkaite, **sel**igmannite, **sta**nnite, **str**omeyerite, **tet**rahedrite, **uma**ngite, **bol**eite, **kin**oite, **cup**rite, **del**afossite, and **ten**orite. 

To shed light on the given classification problem and understand the general similarities of the minerals, they were all grouped in accordance with the Strunz Classes [23] given in Table 1. The first three characters of each mineral name (indicated with bold font in the above list) together with a Strunz Class number constitute short names used in the remainder of this article (with one exception for cornetite being **co0–8**). The exact Strunz Class assignment for each mineral is given in Table S1 in the supplementary data.

The material suppliers were chosen from among different countries and/or geographical regions to differentiate the population of rocks with specific minerals and make the database more versatile. The origins of the samples include six continents, 29 countries, and 65 different regions as follows:

Australia (Kambalda), Austria (Tirol, Steiermark), Belarus (Rhodopien), Chile (Calama, Taltal, Atacama, Chuquicamata), Czechia (Severocesky, Horni Slavkov, Morava, Sredocesky), Democratic Republic Congo (Katanga), France (Corsica), Germany (Aachen, Harz, Hessen, Westerwald, Sauerland, Schwarzwald, Osthessen, Bad Ems, Saxony, Thuringia, Mansfeld), Greece (Laurion), Hungary (Rudabanya), Italy (Udine, Neapel), Japan (Aichi), Kazakhstan (Dzhezkazgan, Majkojyn), Morocco (Agadir, Bou Azzer, Bou Skour), Mexico (Durango), Namibia (Otavi, Kaokoveld), Peru (Huanzala), Poland (Polkowice), Portugal (Estremoz), Russia (Jakutien, Ural), Slovakia (Piesky, Michalovce, Lubjetova), Spain, Switzerland (Grisons, Wallis), Sweden (Gruvasen, Vena), Tajikistan (Mushiston Deposit), UK (Bristol, Cornwall), Ukraine (Nagolny Krjazh), USA (Nevada, Arizona, Montana, Utah, New Mexico, Missouri, California, Michigan, Montana), and Zambia (Kitwe).

**Figure 1:**
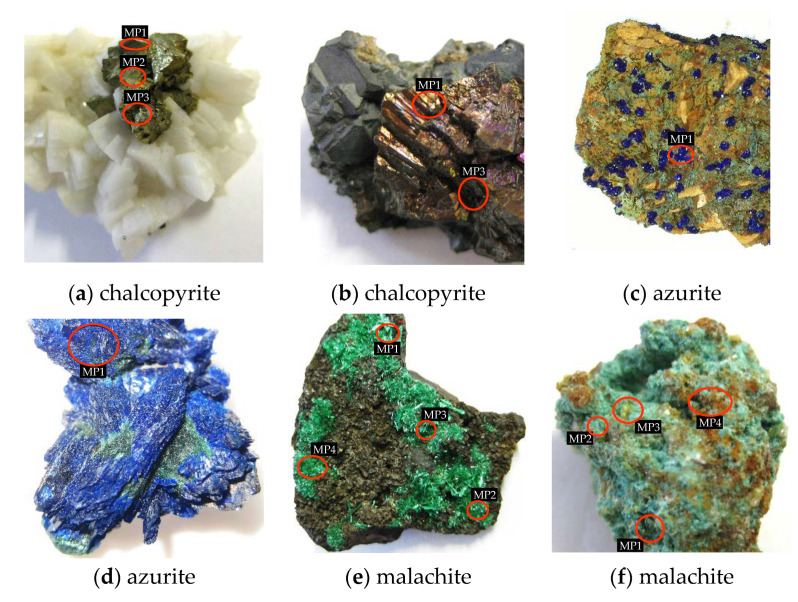
Examples of mineral distribution on the rock samples.

The mineral shapes and sizes attached to the rocks were extremely diverse, from perfect crystals spread on the surface of the base rock to thin layers, often combined with the base rock, with unknown percentage distributions of both. Figure 1 presents examples of such mineral shapes. Examples are for chalcopyrite, azurite, and malachite, where each pair of photos (Figure 1a–f, respectively) shows two cases: (1) a well-built and clear crystal structure without impurities attached to the base rock, and (2) evenly distributed mineral over the rock surface with an unknown percentage mix with the base rock. Moreover, the attached crystal sizes differed from large, as in the case of chalcopyrite (Figure 1a), to very small as in the case of azurite (Figure 1c). 

A total of 127 rock samples were taken into consideration. The statistics for the number of rock samples per mineral are presented in Figure S2 in the supplementary data.

### 2.2. Element Composition

As the chemical bonds are destroyed during the plasma creation, the analysed spectral signal is primarily dependent on the elements that existed in the material before the laser action. All 62 minerals are described in Table S1 in the supplementary data by their empirical element composition taken from [24], where a total of 27 elements were found. Then, based on the elemental composition, an investigation of the similarities between them was conducted with the use of PCA to find theoretically undistinguishable minerals by LIBS. In this way, a new *N*-dimensional (*N* = 27) space was created with new orthogonal variables called principal components (PCs) that describe the dataset sorted from the highest to the lowest variance [25]. Even with such PCA transformation, it was impossible to find good representative PCs to determine how the minerals are distinguishable from each other in a lower dimensional space (i.e., 3D representation).

In that case, *N*-dimensional measures are used to find mineral pairs that are most like each other. For this purpose, the Matlab^®^ pdist function was used with four selected distance measures: Euclidean $d_{st}^{2}={\left(x_{s}-x_{t}\right)}^{T}\left(x_{s}-x_{t}\right)$, City block $d_{st}=\underset{j}{\sum }\left|x_{sj}-x_{tj}\right|$, Chebyshev, $d_{st}=\max_{j}\left\{\left|x_{sj}-x_{tj}\right|\right\}$, and cosine $d_{st}=1-\;\frac{x_{s}^{T}x_{t}}{\left||x_{s}||||x_{t}|\right|}$ [26]. The final computation was an average of those four measures as they gave similar yet different orders of hard to distinguish mineral pairs.

**Figure 2:**
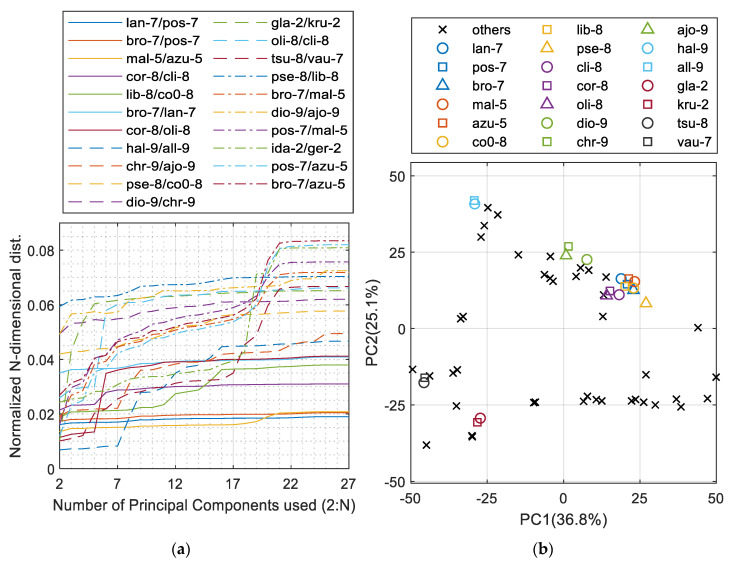
PCA analysis of the mineral dataset based on element composition: (**a**) the *N*-dimensional distance measure between the closest pairs of minerals; (**b**) 2D PCs plot indicating the most similar groups of minerals extracted.

Figure 2a presents the least distinguishable 21 pairs of minerals. The worst cases were the **mal-5** and **azu-5** groups as well as a group consisting of **lan-7**, **pos-7**, and **bro-7**. These two barely reach 2% of the maximum distance between minerals in the PCA space. Figure 2b presents the worst-case groups of minerals extracted from Figure 2a with the other minerals distributed within the space of the first two PCs. Those groups are the already mentioned **lan-7/pos-7/bro-7** and **mal-5/azu-5** as well as **co0-8/lib-8/pse-8**, **cli-8/cor-8/oli-8**, **dio-9/chr-9/ajo-9**, **hal-9/all-9**, **gla-2/kru-2**, and the last one, **tsu-8/vau-7,** which is actually the first pair that shows close similarity above the Strunz Class division; hence, there is no need to search for another similar groups.

**Figure 3:**
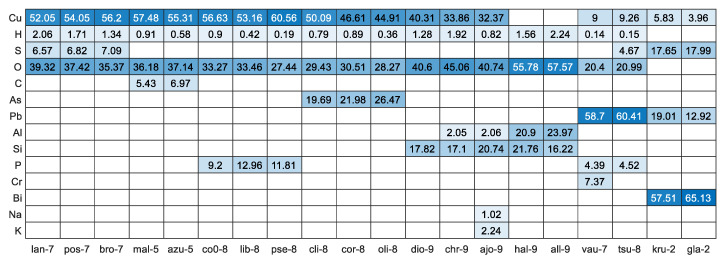
Heatmap (with values) representing elemental composition among the most similar minerals in accordance with the mineral’s empirical formula [24]. The sorting from left to right was performed based on the outcome of Figure 2.

Further analysis of the elemental composition values (Figure 3) clearly indicated that malachite and azurite as well as the group of langite, posnjakite, and brochantite cannot be distinguished from each other. As a result, new mineral labels were created: **a+m** for the **mal-5/azu-5** and **blp** for the other three. These minerals were combined and labelled together for further classification. The full heatmap with all 27 elements and 62 minerals is shown in Figure S3 in the supplementary data.

## 3. Experimental

The measurements were performed using a handheld LIBS device (Z-300, SciAps). The DPSS Nd:YAG laser emits radiation at a wavelength of 1064 nm, with a repetition rate up to 50 Hz, a pulse energy of 5–6 mJ, and a pulse duration of 1–2 ns. The spectrometer covers a range of 190–950 nm. Immediately before and during the measurement, the measuring region was purged with Ar gas to remove ambient air and enhance the LIBS signals. Each specific measurement point (MP) on the rock sample consisted of 64 single shots in an 8 × 8 grid covering an area of roughly 2–4 mm^2^. It is worth noting that the device did not save spectra below a specific intensity threshold. Therefore, in some cases, fewer than 64 spectra were recorded per MP.

**Figure 4:**
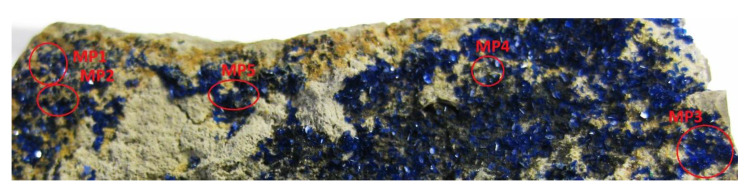
Image of azurite sample measuring points.

The mineral crystals on the surface were uniform, and the area covered by the 8 × 8 grid often extended beyond the crystal of interest. For that reason, all MPs had to be manually labelled. An example of such labelling is shown in Figure 4 and Table 2.

When an MP seems to cover more than 90% of the interesting mineral, it is flagged as a *possible reference*, and can then be used for both training and validation. If not flagged with logic *1* in the Reference column, then the MP remains as *validation only*. In the Coverage column of the description table, a percentage value of the mineral of interest within the 8 × 8 grid is provided. These values are rough assessments (limited to a difference of +/−5%) made just after the LIBS measurement, based on the pattern of laser spots on the sample and descriptions of the minerals of interest made by geologists. This will assist with further automated validation of MPs (because if they reach that percentage value, it means that classification succeeded). Even if the coverage description may in some cases be slightly inaccurate, this does not favour our proposed NTF method as the bias remains the same for all classification algorithms used.

During further classification, the MP is a single entity and the 8 × 8 grid spots will not be separated or analysed exclusively. In total, 458 MPs were recorded, of which 311 were flagged as *possible reference* and 147 as *validation only*. The full statistics of MPs per mineral are given in Figure S1 in the supplementary data.

## 4. Method

### 4.1. Latent Spectrum Extraction

The latent spectrum extraction in its principal form is used to select some underlying (latent) spectra from observed LIBS spectra that are considered as mixtures of multiple latent components generated by multiple spectral sources. The latent spectra are more frequent and have a common pattern across all observed LIBS spectra. In an ideal case, after the extraction, one latent spectrum should resemble an artificial spectrum of the desired source. In practise, we obtain few latent components that approximately represent the true spectra of the analysed minerals.

In the investigated case, each object is probed with several to a dozen measuring points. Thus, each MP contains a maximum of 64 spectra. Let $y_{i_{1},i_{2}}^{\left(m\right)}\in {\mathbb{R}}_{+}^{I_{3}^{\left(m\right)}}$ be the *i*_1_-th spectrum of the *m*-th object, measured in the *i*_2_-th MP. Each MP is assumed to provide $I_{1}^{\left(m\right)}$ spectra, and the number of MPs is equal to $I_{2}^{\left(m\right)}$, where *i*_2_ = 1, …, $I_{2}^{\left(m\right)}$. The space of non-negative numbers is expressed by ℝ_+_. The spectra in each MP are indexed according to a lexicographical order, that is, *i*_1_ = 1, …, $I_{1}^{\left(m\right)}$. The number $I_{1}^{\left(m\right)}$ is usually lower than 64 because some shots that correspond to the spectra of a low variance (below a threshold) must be neglected. The spectral resolution is determined by the number of samples in each spectrum, that is, the number $I_{3}^{\left(m\right)}$. Because the spectral resolution is the same for each observed spectrum, then $\forall m:\;I_{3}=\;I_{3}^{\left(m\right)}.$ We analyse *M* objects, where *m* = 1, …, *M*, assuming that each registered spectrum $y_{i_{1},i_{2}}^{\left(m\right)}$ can be regarded as a superposition of latent spectra that could be pure spectra of analysed minerals (endmembers) or other unwanted or perturbing spectra. The latent spectra for the *m*-th object can be collected into the matrix $U^{\left(m,3\right)}=\left[u_{1}^{\left(m,3\right)},\ldots ,u_{J_{m}}^{\left(m,3\right)}\right]\in {\mathbb{R}}_{+}^{I_{3}\times J_{m}}$, where *J_m_* is the number of latent spectra in the *m*-th object. Considering the above, the spectrum $y_{i_{1},i_{2}}^{\left(m\right)}$ can be expressed by the following superposition rule:

(1) $$y_{i_{1},i_{2}}^{\left(m\right)}=\xi_{i_{1},i_{2,}1}u_{1}^{\left(m,3\right)}+\ldots +\xi_{i_{1},i_{2},J_{m}}u_{J_{m}}^{\left(m,3\right)}=\overset{J_{m}}{\underset{j_{m}=1}{\sum }}\xi_{i_{1},i_{2},j_{m}}u_{j_{m}}^{\left(m,3\right)},$$

where the coefficient $\xi_{i_{1},i_{2},j_{m}}\geq 0$ determines the contribution of the *j_m_*-th latent spectrum to the *i*_1_-th observed spectrum of the *i*_2_-th MP in the *m*-th object. The coefficient $\xi_{i_{1},i_{2},j_{m}}$ can then be factorised as $\xi_{i_{1},i_{2},j_{m}}=u_{i_{1},j_{m}}^{\left(m,1\right)}u_{i_{2},j_{m}}^{\left(m,2\right)}$, where $u_{i_{1},j_{m}}^{\left(m,1\right)}\geq 0$ represents the contribution from the *i*_1_-th shot and $u_{i_{2},j_{m}}^{\left(m,2\right)}\geq 0$ refers to the contribution from the *i*_2_-th MP. Let $u_{j_{m}}^{\left(m,1\right)}=\left[u_{i_{1},j_{m}}^{\left(m,1\right)}\right]\in {\mathbb{R}}_{+}^{I_{1}^{\left(m\right)}}$ be a vector of coefficients $u_{i_{1},j_{m}}^{\left(m,1\right)}$ for *i*_1_ = 1, …, $I_{1}^{\left(m\right)}$, and $u_{j_{m}}^{\left(m,2\right)}=\left[u_{i_{2},j_{m}}^{\left(m,2\right)}\right]\in {\mathbb{R}}_{+}^{I_{2}^{\left(m\right)}}$ for *i*_2_ = 1, …, $I_{2}^{\left(m\right)}$. Sweeping over the indices *i*_1_ and *i*_2_, let $Y^{\left(m\right)}=\left[y_{i_{1},i_{2}}^{\left(m\right)}\right]\in {\mathbb{R}}_{+}^{I_{1}^{\left(m\right)}\times I_{2}^{\left(m\right)}\times I_{3}}$ be a 3-way array (3-modal tensor) created from a set of spectra $\left\{y_{i_{1},i_{2}}^{\left(m\right)}\right\}$ for the *m*-th object. It is thus easy to notice that model (1) takes the form

(2) $$Y^{\left(m\right)}=\overset{J_{m}}{\underset{j_{m}=1}{\sum }}u_{j_{m}}^{\left(m,1\right)}\circ u_{j_{m}}^{\left(m,2\right)}\circ u_{j_{m}}^{\left(m,3\right)},$$

where the symbol ∘ denotes the outer product. Model (2) can be equivalently expressed in the form

(3) $$Y^{\left(m\right)}=J_{m}\times_{1}U^{\left(m,1\right)}\times_{2}U^{\left(m,2\right)}\times_{3}U^{\left(m,3\right)},$$

where $U^{\left(m,1\right)}=\left[u_{1}^{\left(m,1\right)},\ldots ,u_{J_{m}}^{\left(m,1\right)}\right]\in {\mathbb{R}}_{+}^{I_{1}^{\left(m\right)}\times J_{m}}$, $U^{\left(m,2\right)}=\left[u_{1}^{\left(m,2\right)},\ldots ,u_{J_{m}}^{\left(m,2\right)}\right]\in {\mathbb{R}}_{+}^{I_{2}^{\left(m\right)}\times J_{m}}$, $J_{m}\in {\mathbb{R}}_{+}^{J_{m}\times J_{m}\times J_{m}}$ is a superdiagonal identity tensor, and the symbol $\times_{n}$ stands for the tensor-matrix product across the *n*-th mode. 

Note that all factor matrices $\left\{U^{\left(m,1\right)},U^{\left(m,2\right)},U^{\left(m,3\right)}\right\}$ contain only nonnegative numbers, and hence, model (3) can be regarded as the standard non-negative tensor factorisation (NTF) [22], which is a particular case of the CANDECOMP/PARAFAC (CP) decomposition [27,28]. 

Factor $U^{\left(m,3\right)}$ contains *J_m_* latent spectra, and $U^{\left(m,1\right)}$ and $U^{\left(m,2\right)}$ represent the contribution coefficients (concentrations) of the latent spectra to observations across the first and second modes of the tensor $Y^{\left(m\right)}$, respectively. The latent spectra can thus be obtained by performing the NTF of $Y^{\left(m\right)}$, given the assumed number *J_m_*. 

There are numerous computational strategies for NTF, and nearly all of them are based on an alternating optimisation scheme with unfolding imposed on each mode. Model (3) expressed in the unfolded version takes the form

(4) $$Y_{\left(n\right)}^{\left(m\right)}=U^{\left(m,n\right)}{\left({⊙}_{p\neq n}U^{\left(m,p\right)}\right)}^{T},$$

where $Y_{\left(n\right)}^{\left(m\right)}\in {\mathbb{R}}_{+}^{I_{n}^{\left(m\right)}\times \underset{p\neq n}{\prod }I_{p}^{\left(m\right)}}$ is a matrix obtained by the unfolding tensor $Y^{\left(m\right)}$ along its *n*-th mode; where *n* = 1, 2, 3, and the symbol ⊙ stands for the Khatri-Rao product. Note that the system in (4) is considerably overdetermined because $\underset{p\neq n}{\prod }I_{p}^{\left(m\right)}\gg J_{n}$. To alleviate the problem of scaling ambiguity in the NTF, the columns in matrices $U^{\left(m,2\right)}$ and $U^{\left(m,3\right)}$ are normalised to the *unit l*_1_-norm. The system of linear equations in (4) can be solved with numerous linear solvers subject to nonnegativity constraints. In our study, we used the hierarchical alternating least-squares (HALS) algorithm proposed in [29], and then computationally improved in [30]. It belongs to a family of block coordinate descent update algorithms with monotonic convergence and computational complexity of $O\left(NJ_{m}\overset{N}{\underset{n=1}{\prod }}I_{n}^{\left(m\right)}\right)$. The graphical representation of the NTF model is presented in Figure S4 of the supplementary data.

### 4.2. Regression Model Using Latent Spectra 

To estimate the percentage rate of minerals in a newly measured MP, the latent spectra are extracted from the known MPs labelled with the so-called strong reference. We assume that *J_m_* latent spectra are extracted from the *m*-th labelled object using the NTF. The latent spectra after being postprocessed can be regarded as regressors for predicting the percentage of minerals in such an unknown MP. Any unknown spectrum $\tilde{y}\in {\mathbb{R}}_{+}^{I_{3}}$ is assumed to be approximated by a linear regression model,

(5) $$\tilde{y}≅\overset{M}{\underset{m=1}{\sum }}\overset{J_{m}}{\underset{j_{m}=1}{\sum }}\alpha_{j_{m}}^{\left(m\right)}u_{j_{m}}^{\left(m,3\right)},\;\mathrm{where}\;\overset{M}{\underset{m=1}{\sum }}\overset{J_{m}}{\underset{j_{m}=1}{\sum }}\alpha_{j_{m}}^{\left(m\right)}=1.$$

Coefficient $\alpha_{j_{m}}^{\left(m\right)}$ represents the contribution of the *j_m_*-th latent spectrum from the *m*-th object to the unknown spectrum $\tilde{y}$. Let $U=\left[U^{\left(1,3\right)},\ldots ,U^{\left(M,3\right)}\right]\in {\mathbb{R}}_{+}^{I_{3}\times \overset{M}{\underset{m=1}{\sum }}J_{m}}$ and $\alpha =\left[\alpha_{1}^{\left(1\right)},\ldots ,\alpha_{J_{1}}^{\left(1\right)},\alpha_{1}^{\left(2\right)},\ldots ,\alpha_{J_{M}}^{\left(M\right)}\right]\in {\mathbb{R}}_{+}^{\overset{M}{\underset{m=1}{\sum }}J_{m}}$. Coefficient $\alpha_{j_{m}}^{\left(m\right)}$ can be estimated from model (5) by solving the following regularised constrained least-squares problem:

(6) $$\alpha_{*}=\arg\underset{\alpha }{\min}\frac{1}{2}{\left|\left|\tilde{y}-U\alpha \right|\right|}_{2}^{2}+\frac{\lambda }{2}{\left|\left|\alpha \right|\right|}_{2}^{2},\;s.t.\;\alpha \geq 0,\;\mathrm{and}\;{\left|\left|\alpha \right|\right|}_{1}=1,$$

where λ≥0 is a regularisation parameter that controls the overfitting. In this study, problem (6) was solved using the interior-point least-squares algorithms for regularised box-constrained problems, implemented in the function lsqlin in Matlab^®^ 2016a. Note that coefficients in vector $\alpha_{*}$ can also be regarded as unknown percentages of expected minerals in the analysed MP.

### 4.3. Determining the Number of Latent Spectra per Mineral

The last problem to be solved is how to determine the correct (sufficient) *J_m_* number of latent spectra for each *m*-th object. The problem would be trivial if only pure mineral samples were analysed, since only a single latent spectrum may represent the desired mineral. However, in our measurements, impurities in the spectra resulting from base rocks and other minerals will occur. Moreover, the NTF is sensitive to the difference in light intensity distribution between spectrometer channels, which is not constant because the light is propagated inside the device and thus can cause additional perturbations of the desired *m*-th object spectra. In that case, the assumption was made that we will search for *J_m_* latent spectra in which most of them represent the scattered spectra of the desired mineral. The less contributing examples after the so-called self-regression will be labelled as unknown spectra (*U*). 

In such a case, we developed an iterative process in which we start from *J_m_* = 2 (*J_m_* = 1 will be similar to a weighted average of the data) and increment the number until reaching a break loop condition. The loop breaking condition is based on two parameters: level *L* and ratio *R*, which will be set up prior to this procedure.

The *L*-condition is superior and relates to the cumulative contribution given by the sum of coefficients $\left\{\alpha_{j_{m}}^{\left(m\right)}\right\}$ of the first *K* latent spectra ($j_{m}=1,\ldots ,K$), sorted in descending order of their contributions. Note that number *K* cannot be equal to *J_m_*, which means that at least one latent spectrum should be classified as undefined *U*. If the cumulative contribution of *K* latent spectra in a given iterative step is equal to or greater than the *L* value, then the first *K* spectra are assigned as the desired mineral, and the last *J_m_*-*K* spectra are labelled as *U*. Otherwise, *J_m_* is incremented and again the *L*-condition is checked.

If the *L*-condition is satisfied, then the second *R*-condition should be checked. This condition requires the minimum ratio between the last (*K*-th) spectrum assigned as a mineral to the first (*J_m_*-*K*+1) spectrum assigned as *U*. This relation is very important, as for a higher value of *J_m_*, the distribution of latent spectra contributions might end up equal. Therefore, it may be difficult to assess the *U* spectra, as the difference between the last labelled as mineral and first labelled as *U* may be only a few percentage points. If the *R*-condition is not met, *J_m_* is incremented, and the *L*-condition is checked.

After meeting these two (*L* and *R*) conditions, we achieve the solution for which we have the desired level of contributions and the ratio of the contributions of the last mineral spectrum to the first *U* spectrum large enough to assume the *U* spectra are really the unwanted signals. An example of such a loop operation with given *L*/*R* conditions is shown in Figure S5 of the supplementary data.

Obviously, for some combination of *R* and *L* in a given *m*-th object, such a pair of conditions can never be met. To avoid an infinite loop, parameter $J_{m}^{\left(max\right)}$ is introduced, which is the maximum *J_m_* number to be incremented in the loop. If the conditions are not met after $J_{m}=J_{m}^{\left(max\right)},$ then a given pair of *L*/*R* conditions is excluded from consideration for all *m* objects.

**Figure 5:**
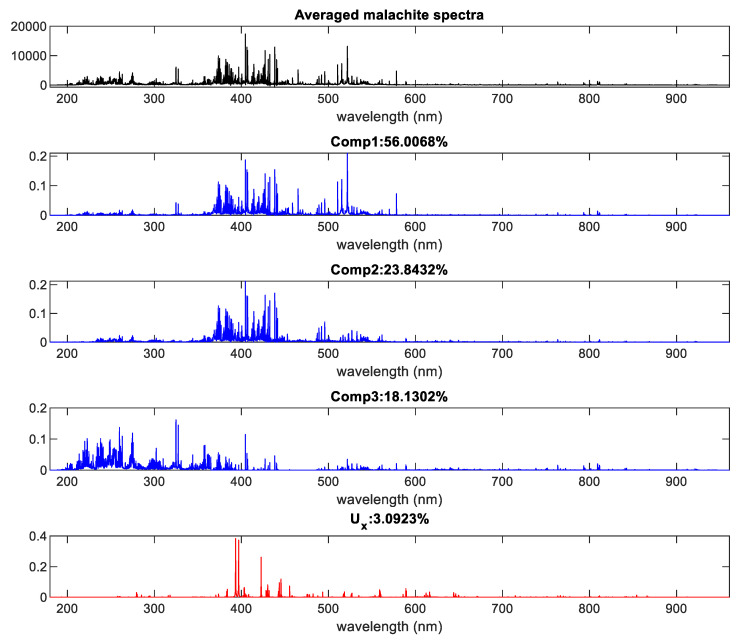
Example of latent spectra extraction. The averaged spectrum originates from all malachite MPs.

Figure 5 presents an example of such latent spectra extraction for malachite with *R* set to 1.8 and *L* to 0.96. In this case, three components were extracted as scattered in the *m*-th object and one as the *U* spectrum.

For the final validation process of a new unknown MP, the scattered *K* latent spectra of each *m*-th object become different regressors. Finally, the predicted percentage contribution for the *m*-th object in the new MP becomes the sum of the single *K* contributions of the *m*-th object scattered latent spectra. The classifying label is then decided on the mineral that has the top percentage contribution.

## 5. Results

### 5.1. Setup

The number of MPs flagged as *validation only* differs among minerals, some do not even have such or have only one. To make the proportion of training and validation data equal among the minerals, some of the MPs flagged as *possible reference* were also moved to be validation data. This new division can be observed in Figure S6 of the supplementary data.

The parameters that were set for our algorithm were a maximum number of latent spectra ($J_{m}^{\left(max\right)}$ = 20), ratios *R* (from 1.1 to 2.0), and levels *L* (from 0.80 to 0.98). The following sections present 10 × 10 heatmaps with classification measures from which we can select the best parameters for the analysis of the confusion matrix. 

Because we deal with an imbalanced dataset, there is a risk of the results being overwhelmed by the outcome of the larger mineral classes. Thus, the metrics of precision and recall are introduced together with their bounding metric called *F*-measure [31]:

(7) $$Precision=\frac{tp}{tp+fp},\;\;Recall=\frac{tp}{tp+fn},\;F=2*\frac{Precision*Recall}{Precision+Recall},$$

where *tp* is the true positive rate, *fp* is the false positive rate, and *fn* is the false negative rate. 

The proposed NTF-based classification also introduces the *U* class (Section 4.3), which should be included in some way in the metrics in (7). For that purpose, we propose to differentiate two cases: *Uin* and *Uex*.

The *Uin* case assumes that we include MPs labelled as *U* in the classification score calculation. However, the metrics in (7) require, among others, true positive rates, which in the case of *U* class, will never exist (*U* is not a true class). This will result in precision and recall being zero all the time and independently from the number of MPs labelled as *U*, the *F*-measure (if calculated) for *U* class will always be zero and, therefore, lower the total *F*-measure for all the classes together. The only way to introduce the *U* class into the *F*-measure, therefore, is assuming that if any MP is going to be assigned as *U* class, it will appear as a false negative value for the original true class (i.e., azurite labelled as *U*).

The *Uex* case totally excludes MPs labelled as *U* from the classification score calculation. This decision was made on the assumption that *U* labels give us the information that the measurement outcome is uncertain and the user should repeat the measurement on such a sample for more confidence (this is not yet an error at that point but restrains us from introducing false positive rates into another class).

Again, as *U* is not a true class, the false positive rates will never exist for it, so, actually, the only difference between *Uin* and *Uex* scores will be held by the recall part of the equation in (7) (the precision will stay the same for both).

The *NaN* values within the heat maps are related to the *L*/*R* pairs with which the algorithm could not find a latent extraction solution for at least one mineral. For improved clarity, each heatmap has the top three *L*/*R* solutions listed in its title.

The result of the proposed algorithm is contrasted with the performance of the Matlab^®^ built-in classification algorithms: SVM (templateSVM: KernelFunction—linear; BoxConstraint—1; standardised input), LDA (fitcdiscr: DiscrimType—linear; standardised input), and KNN (fitcknn: Distance—Euclidean; NumNeighbors—1; DistanceWeight—equal, standardised input) [2]. 

As the confusion matrices in the case of our 59 classes were very large, they were added as supplementary data (Figures S8–S11).

### 5.2. Analysis of Training Data

The first portion of the results is focussed on finding the ideal combination of parameters for our proposed method. To do this, the model was trained and validated using the same training data (Figure S6—supplementary data). This is reasonable, as while the proposed algorithm extracts the latent spectra, it does it within a single mineral class and is unaware of the existence of other classes. Because of this, it is unlikely for the model with so many classes to reach a 100% score even when trained and validated with the same data (which is different in the case of the SVM that reached 100% under the same conditions). However, this gives us an opportunity to revise the model on the basis of training data and determine the best combination of *R* and *L* values for the given dataset.

**Figure 6:**
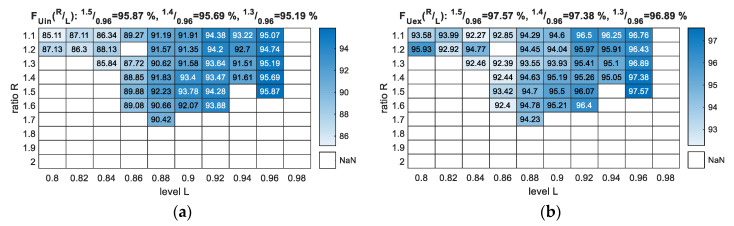
*F*-measure output for the parameterisation of the NTF method with *R* and *L* variables (training data vs. training data): (**a**) *F_Uin_*—scores with *U* class included as an error; (**b**) *F_Uex_*—scores with *U* class excluded from measure.

From Figure 6, we see that the *F*-measure score increased with increasing *L* value. Moreover, it is clear that we reached the parameterisation boundary from each side of the 10 × 10 matrices, as it was impossible to deliver results for *L* equal or greater than 0.98 and *R* equal to 1.8 (or greater). If the *L* values were taken from the range [0.8, 0.82], we had few possible solutions and much lower scores. Finally, it was futile to use *R* values smaller than 1.1 because 1.0 would designate equality. 

The best results for both *F_Uin_* (Figure 6a) and *F_Uex_* (Figure 6b) are a combination of *R* = 1.5 and *L* = 0.96, and those parameters will be selected as a final solution with the validation dataset.

### 5.3. Analysis of Validation Data

For this section, analyses were taken with separate training and validation data, in accordance with Figure S6 in the supplementary data. Although we already selected parameters in the previous section, we decided to perform the same parameterisation of *R* and *L*, resulting in 10 × 10 matrices to verify that the selection was accurate. 

**Figure 7:**
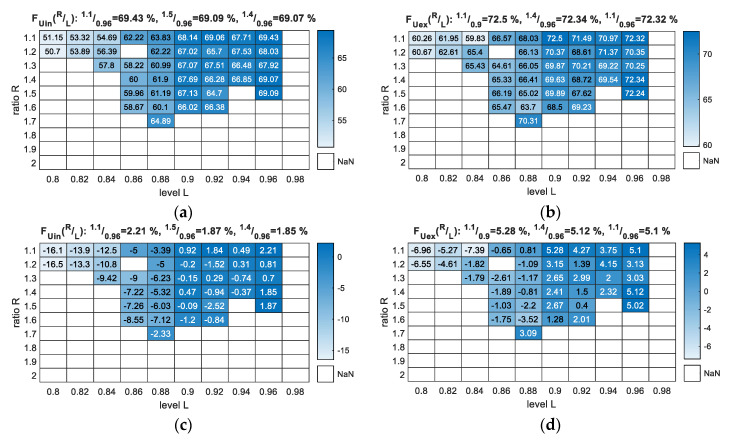
Classification *F*-measure output for the parameterisation of the NTF method with *R* and *L* variables (validation dataset): (**a**) *F_Uin_*—scores with *U* class included as an error; (**b**) *F_Uex_*—scores with *U* class excluded from measure; (**c**) *F_Uin_*—scores with *U* class included as an error in accordance with the best SVM result from Table 3; (**d**) *F_Uex_*—scores with *U* class excluded from measure in accordance with the best SVM result from Table 3.

Similar to Figure 6, the results in Figure 7 indicate (with few exceptions) that the *F*-measure score increased with an increase in *L*. The boundary parameters also remained the same as the learned model, as in the previous section.

Figure 7 presents the *F*-measure results of the parameterisation, but apart from the scores for the *Uin* and *Uex* cases, it also presents the results in comparison to the best selected classifier (Figure 7c,d), which, in our case, was SVM scoring 67.22%. It is important to note here that this was the highest value that was possible to reach for an SVM trying different kernel functions and their parameterisation. In fact, the basic linear SVM scored the best among all SVM variants that was investigated. The additional results for NTF covering the *F*-measure’s partial scores—precision and recall—are presented in Figure S7 in the supplementary data.

Although the best results in the case of Figure 7 were not for *R* = 1.5 and *L* = 0.96, these results were selected as final because they could be foreseen (Section 5.2) and did not differ much from the other high scores, especially in the case of the highest results such as *F_Uex_*.

Table 3 presents the discussed measures obtained for the NTF-based method and three standard classifiers for the analysed validation data. The best built-in solution was SVM, which reached an *F*-measure of approximately 17.5% better than that associated with KNN and LDA. The proposed algorithm reached 1.87% (*Uin*) and 5.02% (*Uex*) of the *F*-measure score with respect to the second-best SVM, followed by increases in every other case where *R_Uex_* had the highest gain of 6.92%. 

The confusion matrices for the built-in classifiers present a trend to seek a host for the more likely hard-to-assess samples. In the case of the best out of three SVM (Figure S8—supplementary data), 23 mineral classes had a 100% score, and the classes that caused the most errors were **blp** (19 false positives) and **a+m** (12 false positives). For LDA (Figure S9—supplementary data), only 11 mineral classes had a 100% score, and the most confusing was **all-5** (16 false positives). The KNN (Figure S10—supplementary data) performed similarly to LDA for the final *F*-measure score, while 13 mineral classes were perfectly assessed, and the classes that caused the most errors were **a+m** (19 false positives) and **ajo-5** (13 false positives). Using the NTF method (Figure S11—supplementary data), we managed to classify the top *F*-measure score and the top 25 mineral classes without error. The *U* class perfectly took the top host position for the hardest to assess samples (17 false positives), followed by **cha-2** (14 false positives) and **cup-4** (8 false positives), which is very reasonable.

The CPU time and disk space usage of the above methods are compared in Table S12 in the supplementary data. It is clear from this table that the proposed NTF-based method requires less disk space and needs half the time for data validation compared to the competing SVM.

### 5.4. Example of Mineral Contribution for Selected MPs

**Figure 8:**
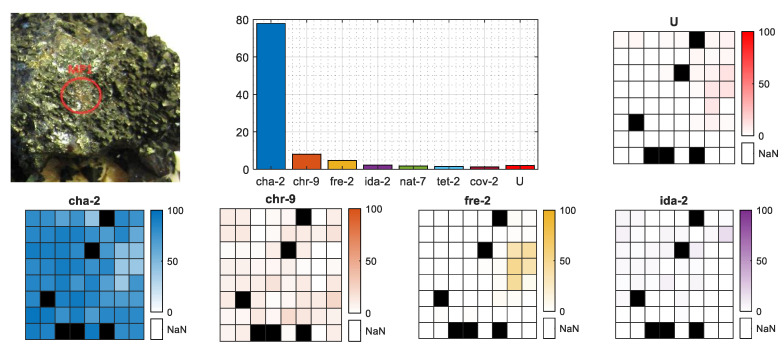
Mineral contribution for selected MP of chalcopyrite sample. Bar chart presents the total percentage rate of minerals per MP, the image indicates the place of measurement on the sample surface and the heatmaps present the geometrical distribution of the four main minerals and unknown class within 8 × 8 shooting grid. Black fields are missing spectra.

The regression method proposed, apart from classifying the MPs, also gives the mineral percentage contribution followed by their geometrical distribution within the 8 × 8 shooting grid. Figure 8 presents the results for MP1 from the selected chalcopyrite sample. The bar plot presents the single contributions of the regressors (*J_m_* latent spectra) summed for each mineral. In this MP case, the top count goes to chalcopyrite (almost 80%), leaving all the other minerals far behind, so the given classification label is correct. The geometrical distributions of the four main minerals (*m*-th objects) and the unknown class were equal within the 8 × 8 grid. Both can be confirmed with the MP photo, as the surface looks like an equal mineral distribution without any sign of the base rock.

**Figure 9:**
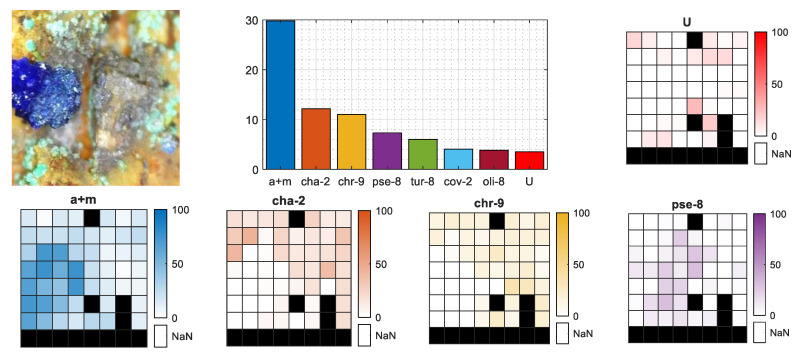
Mineral contribution for selected MP of azurite sample. Bar chart presents the total percentage rate of minerals per MP, the image indicates the place of measurement on the sample surface and the heatmaps present the geometrical distribution of the four main minerals and unknown class within 8 × 8 shooting grid. Black fields are missing spectra.

However, as mentioned in Section 2, there were also samples that did not have a consistent mineral distribution on the surface, and the crystal size was even smaller than the 8 × 8 shooting grid. An example of such a situation is the selected azurite sample presented in Figure 9. From the photo, we observe that the azurite crystal actually covers less than one-quarter of the visible, burned in laser pattern. This fact is clearly visible in the mineral distribution heatmaps of **a+m**, **cha-2**, and **chr-9**, where in the case of the proper azurite (**a+m**) class in the bottom-left quarter, we observe a higher contribution of that mineral, while in the same area on the **cha-2** and **chr-9** heatmaps, there is almost zero contribution. The total sum of **a+m** contribution on the bar plot is almost 30%, which perfectly covers one-quarter of the crystal that fits within the 8 × 8 shooting grid plus some average error of the latent spectra contribution.

In both cases, as the classification labels were correctly set, the *U* class did not play an important role in the final percentage rate within those MPs. Both MPs are missing some spectra.

## 6. Conclusions

In this research, we hypothesised that it is possible to extract artificial (latent) spectra for each of the investigated minerals and use them as predictors in a linear regression model. For this purpose, NTF was used. Because of the heterogeneity of the mineral samples and weak reproducibility of the spectra acquired with the use of a handheld LIBS device, the procedure for proper latent spectra selection was proposed. In such a procedure, a parameterisation of ratio *R* and level *L* variables is required. The results show that these variables can be limited, with high accuracy, to one selection by performing validation with the use of the same data as for the model training. 

The NTF-based classification performed well, reaching higher *F*-measure scores of around 1.9% (when the Unknown class was included in the measures) and around 5.0% (when the Unknown class was excluded from the measures), both in accordance with the best SVM classifier. The standard methods seem to find a host class for the hardest-to-assess samples, so using the method that already contains an Unknown class inside its model was even more reasonable here. 

In addition to the output labelling required for classification purposes, a percentage contribution of the minerals within measuring points is given. Such additional information on the MPs allows the creation of 2D mapping of the shooting area 8 × 8 grids with a smooth distribution of the mineral classes among them.

The final regression model can be stored using low disk space, and the regression function is not as memory- and CPU-intensive (compared to the commonly used classifiers SVM, LDA, and KNN). Such a model may even be implemented in mobiles and other handheld LIBS devices and thus increase their functionality as fast, on-site mineral analysers.

Future research will be devoted to applying the method to minerals other than those containing copper as their primary element and verifying its universal usability. The NTF was confirmed as a method for extracting artificial spectra for the mineral classes and was successfully used as a regressor in a linear model. 

## Figures and Tables

**Table 1:** List of mineral classes of analysed samples (based on original Strunz Class [23]).

Mineral Class Name	Class Number C
Sulfides	2
Halides	3
Oxides and Hydroxides	4
Carbonates and Nitrates	5
Sulfates	7
Phosphates	8
Silicates	9

**Table 2:** Example description table of the sample shown in Figure 4.

MP	Mineral	Reference	Coverage
1	azurite	0	80
2	azurite	0	20
3	azurite	1	90
4	azurite	0	30
5	azurite	0	60

**Table 3:** Classification measures of the validation dataset with the use of analysed methods. Brackets indicate the NTF accuracy gain in comparison to the best SVM classifier.

Measure	SVM	KNN	LDA	NTF_Uin_	NTF_Uex_
**Precision (%)**	71.76	51.77	52.01	74.74 (+2.98)	74.74 (+2.98)
**Recall (%)**	68.48	54.97	54.16	70.13 (+1.65)	75.40 (+6.92)
***F*-Measure (%)**	67.22	49.48	49.77	69.09 (+1.87)	72.24 (+5.02)

## Supplementary Materials

The following are available online at https://www.mdpi.com/1424-8220/20/18/5152/s1, Table S1: detailed list of 62 minerals used in research; Figure S1: the number of measurement points (MPs) taken per mineral together with their partitions into *possible reference* and *validation only* ones; Figure S2: the number of different (exclusive) rock samples on which MPs were taken—listed per mineral; Figure S3: heatmap representing elements composition among all 62 minerals in accordance with the mineral’s empirical formula [24]; Figure S4: NTF model; Figure S5: example of *J_m_* number of latent spectra decision for one *m*-th object; Figure S6: dataset partitioning of the training and validation data using some of the *possible reference* MPs as validation data to evenly match the dataset among mineral classes; Figure S7: classification precision and recall output for the parametrisation of the NTF method with *R* and *L* variables (validation dataset), Figure S8: confusion matrix of SVM classifier for validation dataset; Figure S9: confusion matrix of KNN classifier for validation dataset; Figure S10: confusion matrix of LDA classifier for validation dataset; Figure S11: confusion matrix of NTF based classifier for validation dataset; Table S12: CPU time and disk space usage of the compared methods.
