# Supplementary material for: Classification of Copper Minerals by Handheld Laser-Induced Breakdown Spectroscopy and Nonnegative Tensor Factorisation
Source: Sensors (Basel). 2020 Sep 9;20(18):5152. doi: 10.3390/s20185152 (PMC7570571; doi:10.3390/s20185152)
Supplement: Supplementary file 1 [file sensors-20-05152-s001.pdf]

sensors

MDPI

Supplementary data

Table S1. (part 1) Detailed list of 62 minerals used in research. C refers to Class (Table 1). Bold font in names refers to the short names used in figures.

Mineral name	C	Empirical formula	Mineral name	C	Empirical formula
**algo**donite	2	Cu_6As	**azur**ite	5	$Cu_3(CO_3)_2(OH)_2$
**anti**monpearceite	2	$Ag_{12}Cu_4Sb_{1.5}As_{0.5}S_{11}$	**mal**achite	5	$Cu_2(CO_3)(OH)_2$
**bor**nite	2	$Cu_5Fe^{2+}S_4$	**broch**antite	7	$Cu_4(SO_4)(OH)_6$
**bour**nonite	2	$PbCuSbS_3$	**copi**apite	7	$Fe^{2+}Fe^{3+}_4(SO_4)_6(OH)_2 \bullet 20(H_2O)$
**chal**copyrite	2	$CuFe^{2+}S_2$	**dev**illine	7	$CaCu_4(OH)_6(SO_4)_2 \bullet 3(H_2O)$
**col**usite	2	$Cu_{12.5}V^{5+}As_{1.5}Sb_{0.9}Sn_{0.6}Ge_{0.3}S_{16}$	**forn**acite	7	$Pb_2Cu(AsO_4)(CrO_4)(OH)$
**cov**ellite	2	CuS	**lang**ite	7	$Cu_4(SO_4)(OH)_6 \bullet 2(H_2O)$
**dig**enite	2	Cu_9S_5	**nak**auriite	7	$Mn^{2+}_4Ni_3Cu(SO_4)_4(CO_3)(OH)_6 \bullet 48(H_2O)$
**en**argite	2	Cu_3AsS_4	**natro**chalcite	7	$NaCu_2(SO_4)_2(OH) \bullet (H_2O)$
**frei**bergite	2	$Ag_{7.2}Cu_{3.6}Fe^{2+}_{1.2}Sb_3AsS_{13}$	**osa**rizawaite	7	$PbCuAl_2(SO_4)_2(OH)_6$
**ger**manite	2	$Cu_{26}Fe^{2+}_4Ge_4S_{32}$	**pos**njakite	7	$Cu_4(SO_4)(OH)_6 \bullet (H_2O)$
**glad**ite	2	$PbCuBi_5S_9$	**vau**quelinite	7	$Pb_2Cu(CrO_4)(PO_4)(OH)$
**ida**ite	2	$Cu_5Fe^{2+}S_6$	**art**hurite	8	$CuFe^{3+}_2 2(AsO_4)_{0.7}2(PO_4)_{0.2}2(SO_4)_{0.1}O_{1.5}(OH)_{0.5} \bullet 4(H_2O)$
**jas**kolskiite	2	$Pb_{2.2}Cu_{0.2}Sb_{1.2}Bi_{0.6}S_5$	**chen**evixite	8	$Cu_2Fe^{3+}_2(AsO_4)_2(OH)_4 \bullet (H_2O)$
**kru**pkaite	2	$PbCuBi_3S_6$	**cli**noclase	8	$Cu_3(AsO_4)(OH)_3$
**sel**igmannite	2	$PbCuAsS_3$	**con**ichalcite	8	$CaCu(AsO_4)(OH)$
**sta**nnite	2	$Cu_2Fe^{2+}SnS_4$	cornetite (**co0**)	8	$Cu_3(PO_4)(OH)_3$
**stro**meyerite	2	$CuAgS$	**cor**nubite	8	$Cu_5(AsO_4)_2(OH)_4 \bullet (H_2O)$
**tet**rahedrite	2	$Cu_9Fe^{2+}_3Sb_4S_{13}$	**des**cloizite	8	$PbZn(VO_4)(OH)$
**uma**ngite	2	Cu_3Se_2	**duf**tite	8	$PbCu(AsO_4)(OH)$
**bol**eite	3	$KAg_9Cu_{24}Pb_{26}Cl_{62}(OH)_{48}$	**lib**ethenite	8	$Cu_2(PO_4)(OH)$
**kin**oite	3	$Ca_2Cu_2Si_3O_{10} \bullet 2(H_2O)$	**mott**ramite	8	$PbCu(VO_4)(OH)$
**cup**rite	4	Cu_2O	**oli**venite	8	$Cu_2(AsO_4)(OH)$
**del**afossite	4	$CuFe^{3+}O_2$	**par**nauite	8	$Cu_9(AsO_4)_2(SO_4)(OH)_{10} \bullet 7(H_2O)$
**ten**orite	4	CuO	**pse**udomalachite	8	$Cu_5(PO_4)_2(OH)$
**del**afossite	4	$CuFe^{3+}O_2$	**tur**quoise	8	$CuAl_6(PO_4)_4(OH)_8 \bullet 4(H_2O)$

Table S1. (part 2) Detailed list of 62 minerals used in research. C refers to Class (Table 1). Bold font in names refers to the short names used in figures.

Mineral name	C	Empirical formula
**ric**helsdorfite	8	$Ca_2Cu_5Sb(AsO_4)_4Cl(OH)_6{\bullet}6(H_2O)$
**tsu**mebite	8	$Pb_2Cu(PO_4)(SO_4)(OH)$
**tyr**olite	8	$CaCu_5(AsO_4)_2(CO_3)(OH)_4{\bullet}6(H_2O)$
**ajo**ite	9	$K_{2.25}Na_{1.75}Cu_{20}Al_3Si_{29}O_{76}(OH)_{16}{\bullet}8(H_2O)$
**all**ophane	9	$(Al_2O_3)(SiO_2)_{1.3}{\bullet}2.5(H_2O)$
**chr**ysocolla	9	$Cu_{1.75}Al_{0.25}H_{1.75}(Si_2O_5)(OH)_4{\bullet}0.25(H_2O)$
**cre**aseyite	9	$Pb_2Cu_2Fe^{3+}_{1.75}Al_{0.25}Si_5O_{17}{\bullet}6(H_2O)$
**dio**ptase	9	$CuSiO_2(OH)_2$
**hal**loysite	9	$Al_2Si_2O_5(OH)_4$
**pla**ncheite	9	$Cu_8Si_8O_{22}(OH)_4{\bullet}(H_2O)$
**ves**uvianite	9	$Ca_{10}Mg_2Al_4(Si_2O_7)_2(SiO_4)_5(OH)_4$

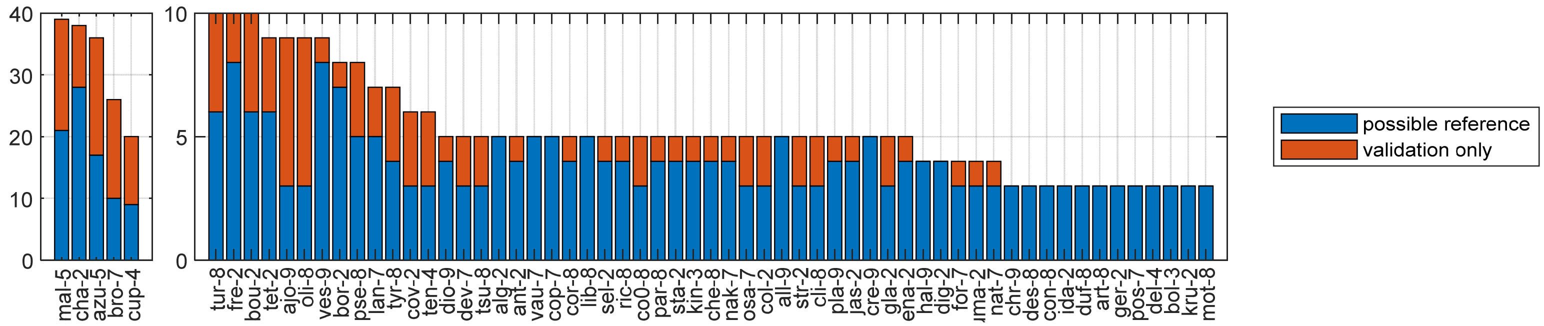

Figure S1. The number of measurement points (MPs) taken per mineral together with their partitions into *possible reference* and *validation only* ones.

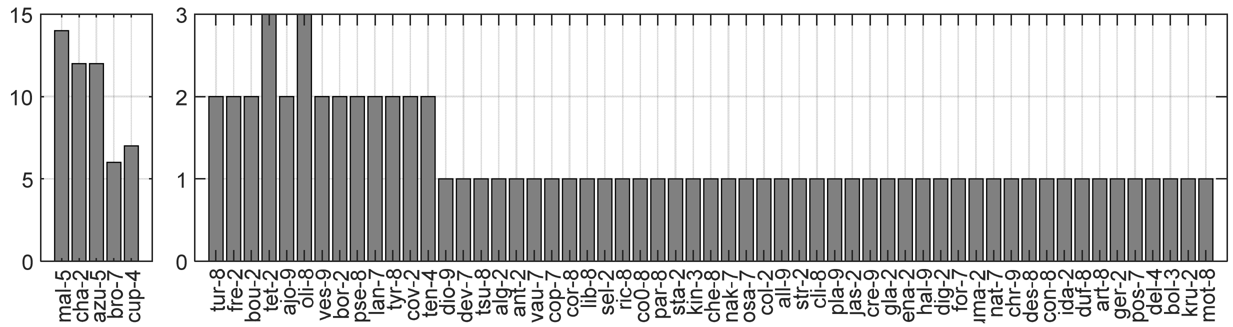

Figure S2. The number of different (exclusive) rock samples on which MPs were taken – listed per mineral.

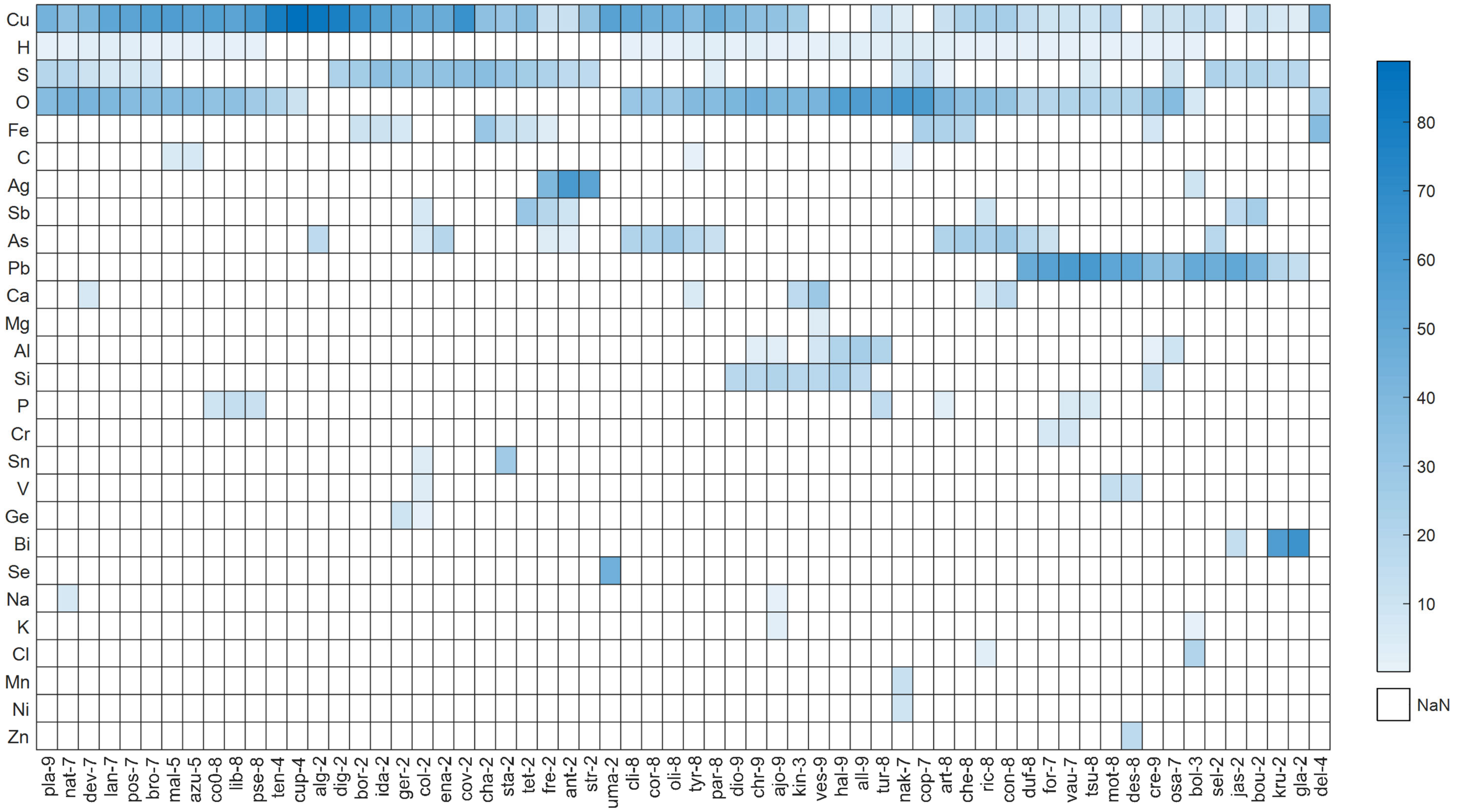

Figure S3. Heatmap representing elements composition among all 62 minerals in accordance to mineral's empirical formula [24]. The sorting from left to right was done based on the shortest *N*-dimensional distance between each pair of minerals where the first **pla-9** is the closest to the origin of PCA space.

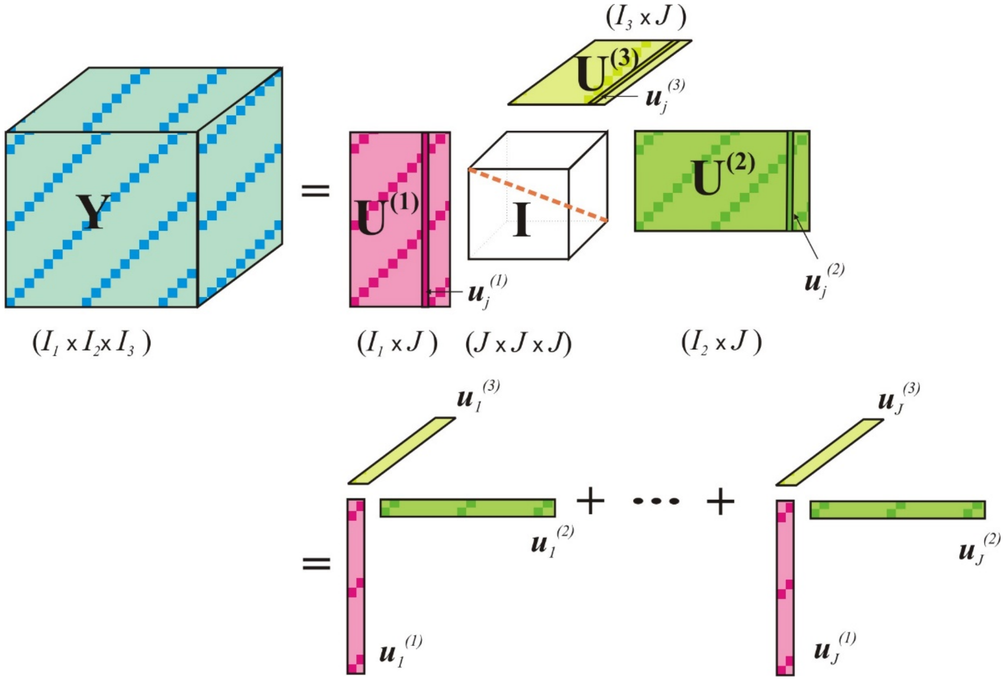

Figure S4. NTF model: a nonnegative 3-way tensor Y is represented by a tensor-matrix product of the latent component matrices $\{U^{(1)}, U^{(2)}, U^{(3)}\}$ with an identity tensor I or equivalently by a sum of J rank-1 tensors obtained by an outer product of the latent components.

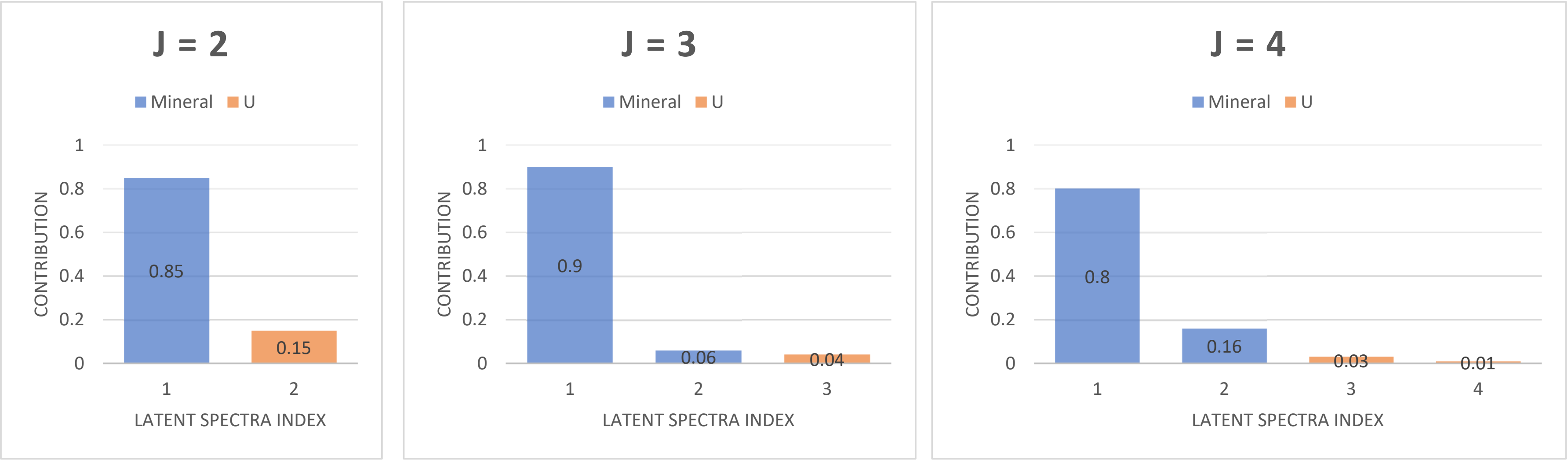

(a) (b) (c)

Figure S5. Example of J_m number of latent spectra decision for one m-th object. The L is set to 0.95 and R to 2.0 and maximum latent division $J_m^{(max)}$ to 10:

a) First iteration - J_m equals 2. Assuming one spectra always needs to be left as U then the sum of coefficients $\{\alpha_{j_m}^{(m)}\}$ of the first K latent spectra (K equals only 1 in that case) covers only 0.85 and the L-condition is not met – J_m is incremented.

b) Second iteration - J_m equals 3. Assuming one spectrum always needs to be left as U then the minimum sum of coefficients $\{\alpha_{j_m}^{(m)}\}$ of the first K latent spectra that meets L is 0.96 for K=2, so the L-condition is met. Spectra 1 and 2 are assigned as mineral while spectrum 3 is assigned as U. Then the second R parameter is checked. The ratio between last mineral (blue) and first U spectrum (orange) is 1.5 which does not meet the R value – hence J_m is incremented. It is worth noting here that the difference between contributions of the coefficients of second spectrum (6 percent) and third spectrum (4 percent) compared to the first spectrum (90 percent) makes it unclear if maybe both second and third spectra are rather U. But in that case, the L-condition is not met, so a given pair of L/R will not work for $J_m = 3$.

c) Third iteration - J_m equals to 4. Assuming again that one spectrum always needs to be left as U, then the minimum sum of coefficients $\{\alpha_{j_m}^{(m)}\}$ of the first K latent spectra that meets the L minimum contribution is 0.96 for K=2, so the L-condition is met. Spectra 1 and 2 are assigned as mineral while spectra 3 and 4 are assigned as U. Then the second R-condition is checked. The ratio between the last mineral (blue) and the first U spectrum (orange) is 5.3 which meets the minimum R-condition = 2.0. The loop is broken and the solution for the m-th object with $J_m = 4$ is given. In such a case, we have two spectra that are assigned as mineral (and later in regression will be labeled as m-th object) and two spectra with minor contribution labeled as U.

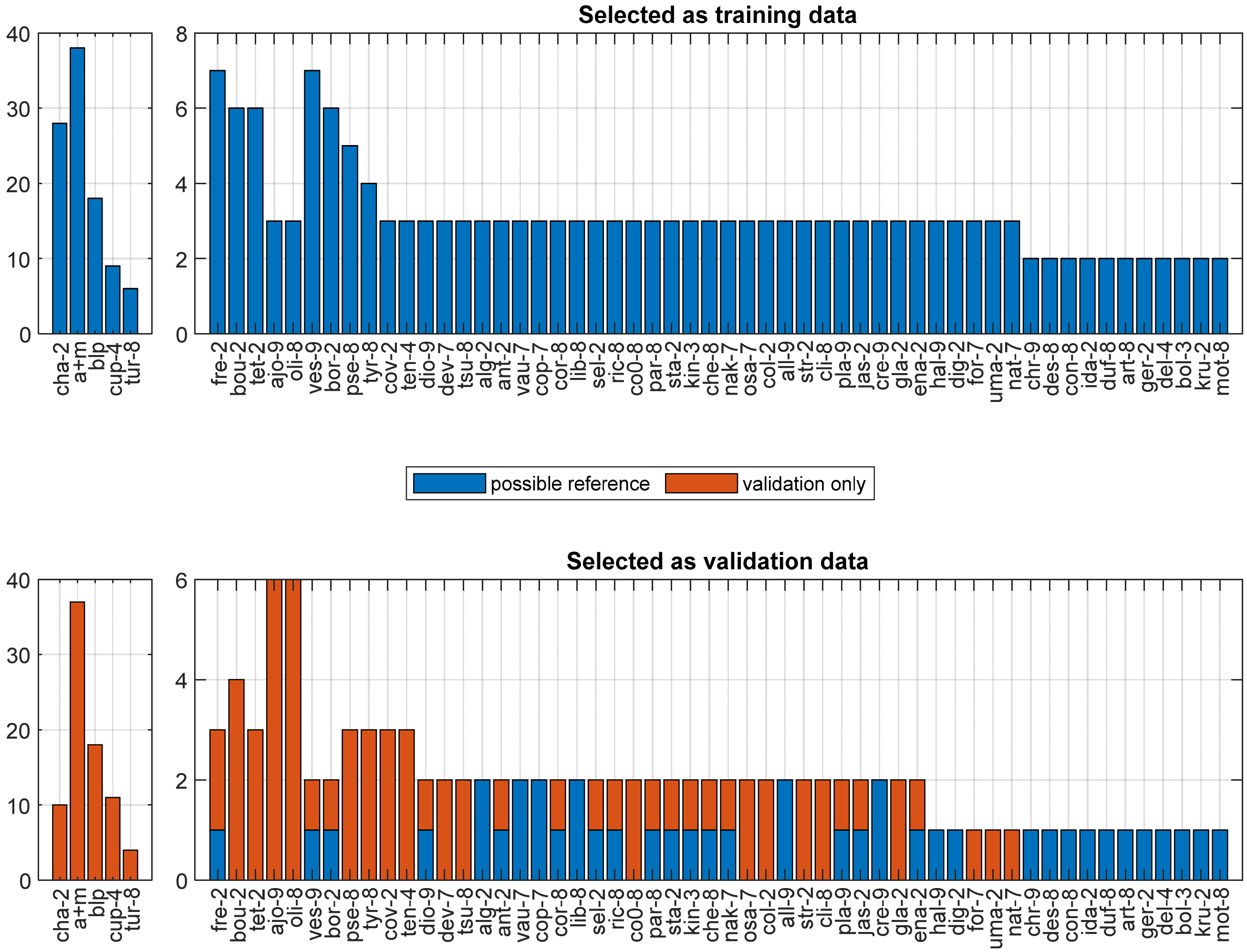

Figure S6. Dataset partitioning of the training and validation data using some of the *possible reference* MPs as validation data to evenly match the dataset among mineral classes (Section 5.3).

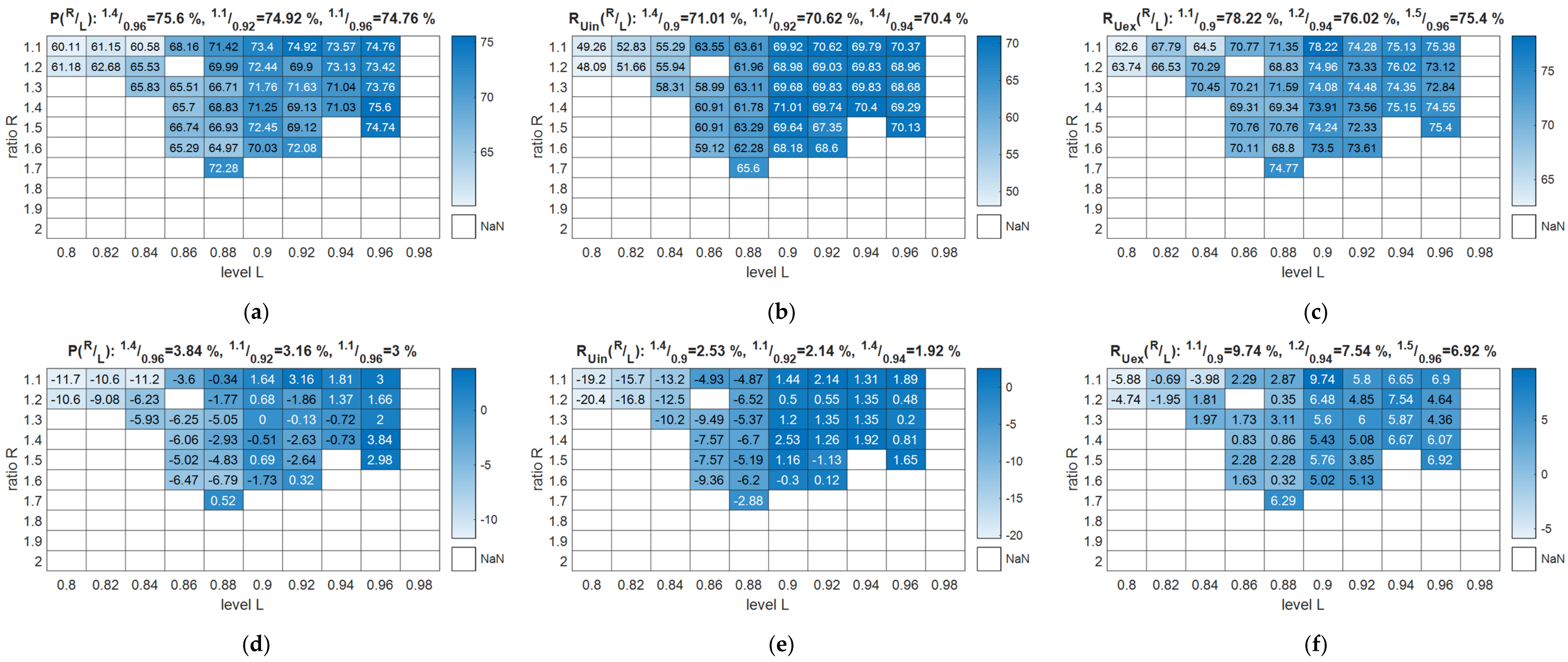

Figure S7. Classification precision and recall output for the parametrisation of the NTF method with *R* and *L* variables (validation dataset): (**a**) *P* – precision scores; (**b**) R_{Uin} – recall scores with the *U* class included as an error; (**c**) R_{Uex} – recall scores with the *U* class excluded from the measure; (**d**) *P* – precision scores in accordance with the best SVM result from Table 3; (**e**) R_{Uin} – recall scores with the *U* class included as an error in accordance with the best SVM result from Table 3; (**f**) R_{Uex} – recall scores with the *U* class excluded from the measure in accordance with the best SVM result from Table 3.

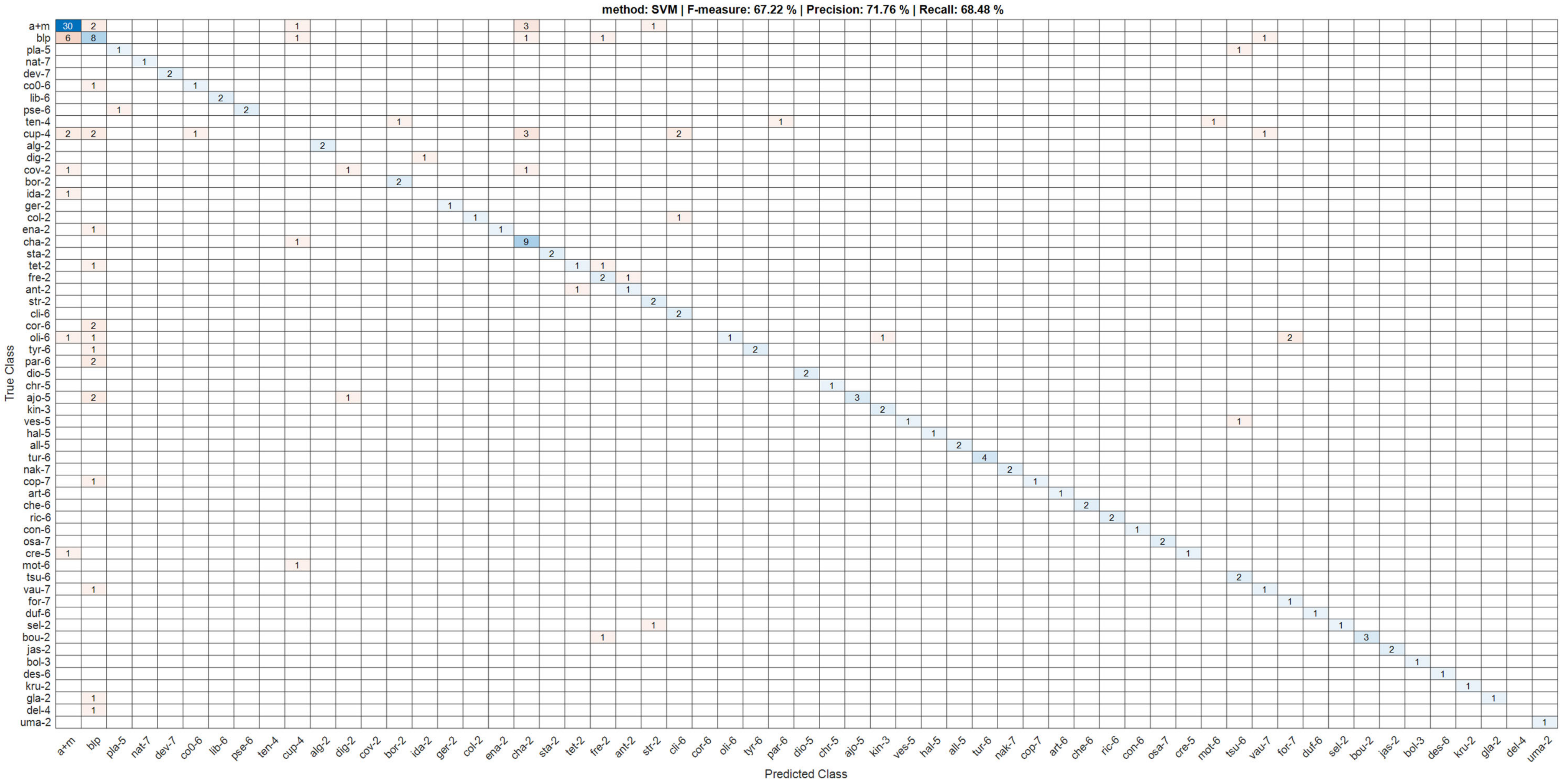

Figure S8. Confusion matrix of the SVM classifier for validation dataset (Section 5.3).

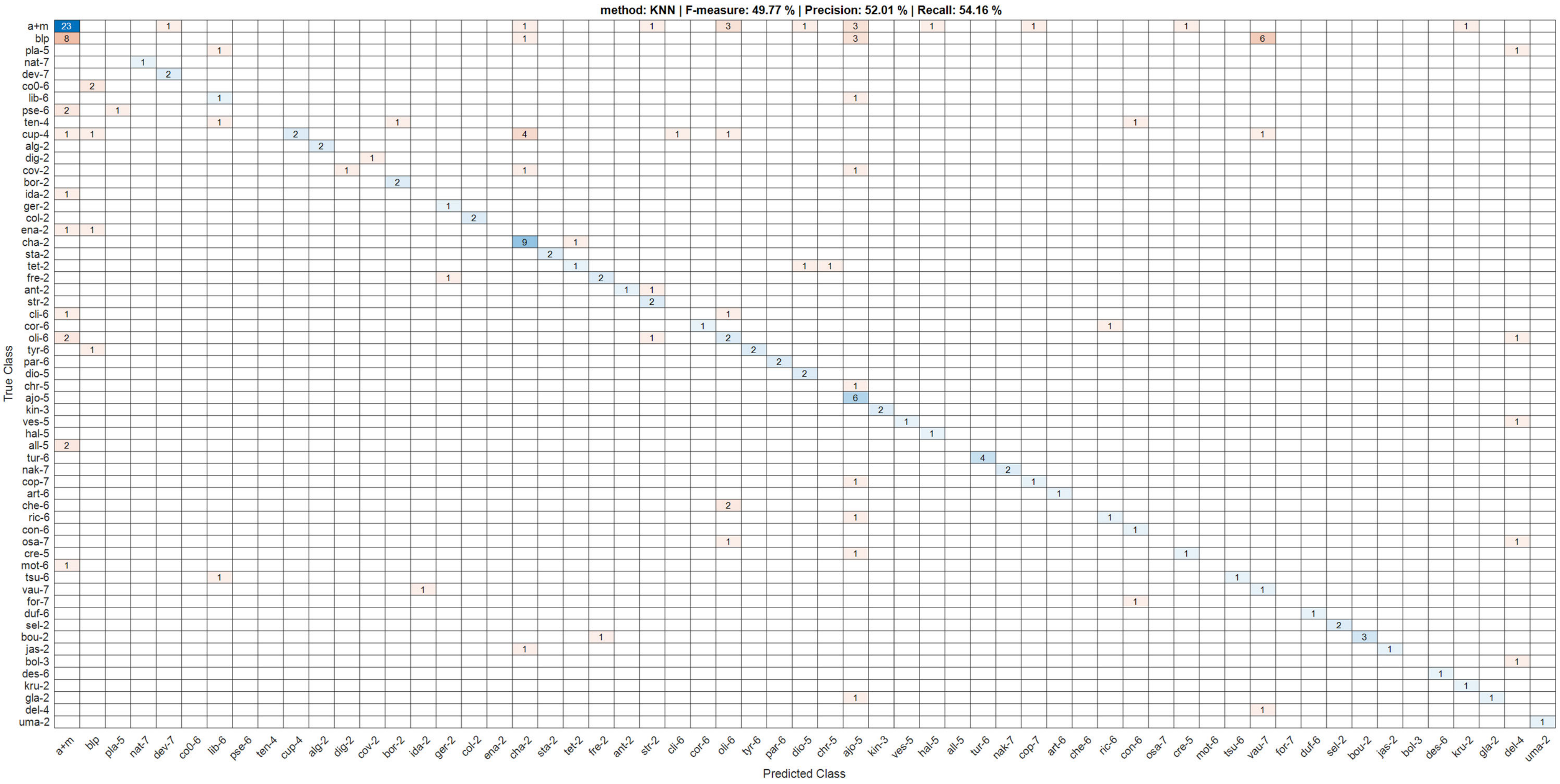

Figure S9. Confusion matrix of the KNN classifier for validation dataset (Section 5.3).

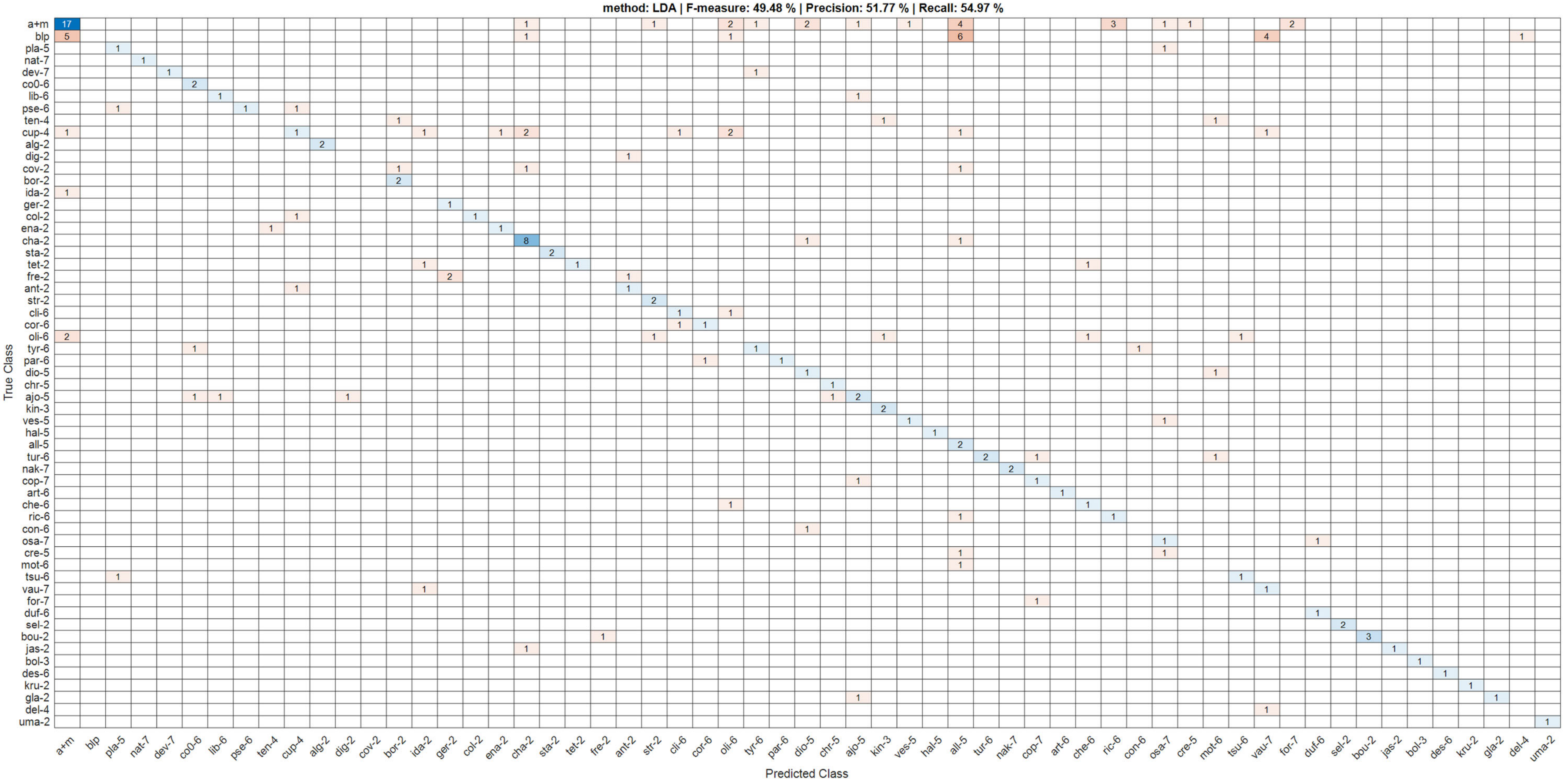

Figure S10. Confusion matrix of the LDA classifier for validation dataset (Section 5.3).

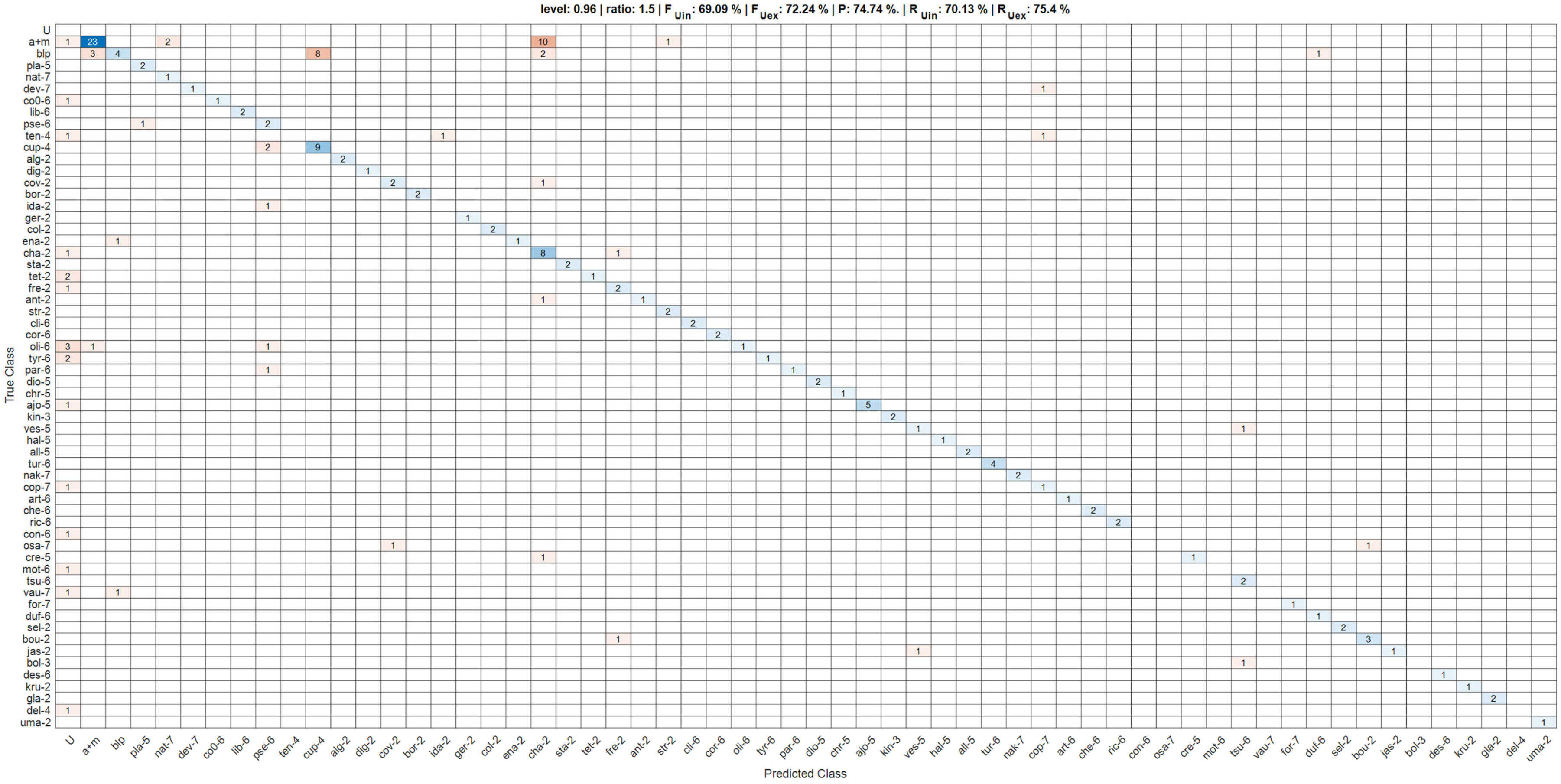

Figure S11. Confusion matrix of the NTF based classifier for validation dataset (Section 5.3).

Table S12. CPU time and disk space usage of the compared methods. The desktop hardware was Intel i7-4790k (4.00GHz) with 32 GB RAM.

Method	CPU learning time (s)	CPU validation time for 188 MPs (s)	Disk space usage (MB)
**SVM**	1274.50	1134.26	1378.59
**LDA**	199.52	27.23	1440.37
**KNN**	4.26	462.27	964.46
**NTF**	1389.22	574.65	25.19
